# Primary diffuse Rosai-Dorfman disease in central airway: a case report and literature review

**DOI:** 10.1186/s12890-023-02363-1

**Published:** 2023-03-10

**Authors:** Lei Wu, Yan Zhang, Xiaoya Guo, Xing Tang, Keke Xin, Li Yang, Zhe Wang, Wen Jian, Feng Zhao

**Affiliations:** 1 Department of Respiratory and Critical Care Medicine, Xijing Hospital, Fourth Military Medical University, No. 127 West Changle Road, Xi’an, 710032 Shaanxi China; 2 Department of Radiology, Xijing Hospital, Fourth Military Medical University, No. 127 West Changle Road, Xi’an, 710032 Shaanxi China; 3 Department of Nuclear Medicine, Xijing Hospital, Fourth Military Medical University, No. 127 West Changle Road, Xi’an, 710032 Shaanxi China; 4 Department of Pathology, Xijing Hospital and Basic Medicine School, Fourth Military Medical University, No. 127 West Changle Road, Xi’an, 710032 Shaanxi China

**Keywords:** Rosai-Dorfman disease, Central airway, Transbronchial resection

## Abstract

**Background:**

Rosai-Dorfman disease (RDD) is a rare benign non-langerhans cell histiocytosis, mainly involving lymph nodes and skin. It is even rarer occurring only in central airway of lung and in diffuse form. Central airway RDD is similar to malignant tumor in imaging by radiological method and in bronchoscopy features. It is difficult to differentiate it from primary airway malignant tumor and to diagnose correctively in time.

**Case presentation:**

Here we present a rare case of 18-year-old male diagnosed with primary diffuse RDD in central airway. Although the features examined by enhanced chest computed tomography, positron emission tomography/computed tomography, diffusion-weighted imaging of enhanced chest MRI and bronchoscopy indicate to be malignant tumor, the patient was definitely confirmed by multiple transbronchial biopsies and immunohistochemistry. After two transbronchial resections, the patient's symptoms of paroxysmal cough, whistle sound and shortness of breath were significantly reduced, as well as the airway stenosis was significantly improved. After 5 months of follow-up, the patient had no symptoms and the central airway were unobstructed.

**Conclusions:**

Primary diffuse RDD in central airway is characterized by intratracheal neoplasm, which is usually suspected as malignant tumor according to radiological image and bronchoscopy. Pathology and immunohistochemistry are necessary for definite diagnosis. Transbronchial resection is effective and safe for patients with primary diffuse RDD in central airway.

## Background

Rosai-Dorfman disease (RDD), known as sinus histiocytosis with massive lymphadenopathy, is a rare benign non-langerhans cell histiocytosis, mainly involving in lymph nodes (30–50%) and skin (50%)[1]. It is even rarer to happen only in central airway of lung and in diffuse form. The purpose of this case presentation is to improve the clinicians' understanding of primary diffuse RDD in central airway of lung by reviewing the detailed diagnosis and treatment process of a patient in our hospital, so as to improve the patient's prognosis in future.

### Case presentation

The patient was an 18-year-old male. From May 2021, he had a paroxysmal cough, whistle sound during sleep and shortness of breath. Enhanced chest computed tomography (CT) in a local hospital showed neoplasms on the trachea and the opening of the right main bronchus respectively on June 1, 2021. Pulmonary function test showed forced expiratory volume in 1 s (FEV_1_)/forced vital capacity (FVC) was 0.63, FVC predicted was 56.29%, diffusing capacity for carbon monoxide (DLCO) predicted was 71.46%, maximal voluntary ventilation (MVV) predicted was 40.6%, and bronchial dilation test was negative. Flexible fiber scope showed a neoplasm in the middle 1/3 segment of the trachea. The length of the neoplasm was about 1.5 cm, and had wide base and papillary top, resulting in about 40% of airway stenosis. Another neoplasm was found in the opening of the right main bronchus, resulting in about 90% of airway stenosis. The two lesions were independent and the tracheal mucosa between these two lesions was smooth and intact. The flexible fiber scope could enter along the gap, and the ridge between the right upper bronchus and intermediate bronchus was widened. Biopsy of the lesion at the opening of the right main bronchus was performed. A large number of lymphocytes were found infiltrated in the lesion by hematoxylin and eosin (HE) staining. Only a small number of Ki-67 positive cells could be found in the lesion. In addition to this, we also found that TTF-1 (−), Syn (−), and LCA ( +) in lymphocyte, CD3 ( +) in T lymphocyte, CD20 ( +) in B lymphocyte by immunohistochemistry. In this patient, left nasal polypectomy was performed in August 2020 and postoperative pathology showed left nasal polyp with a little eosinophil infiltration. The patient had no allergic history, no cancer in his family history, no smoking and drinking, no special occupational exposure.

**Fig. 1:**
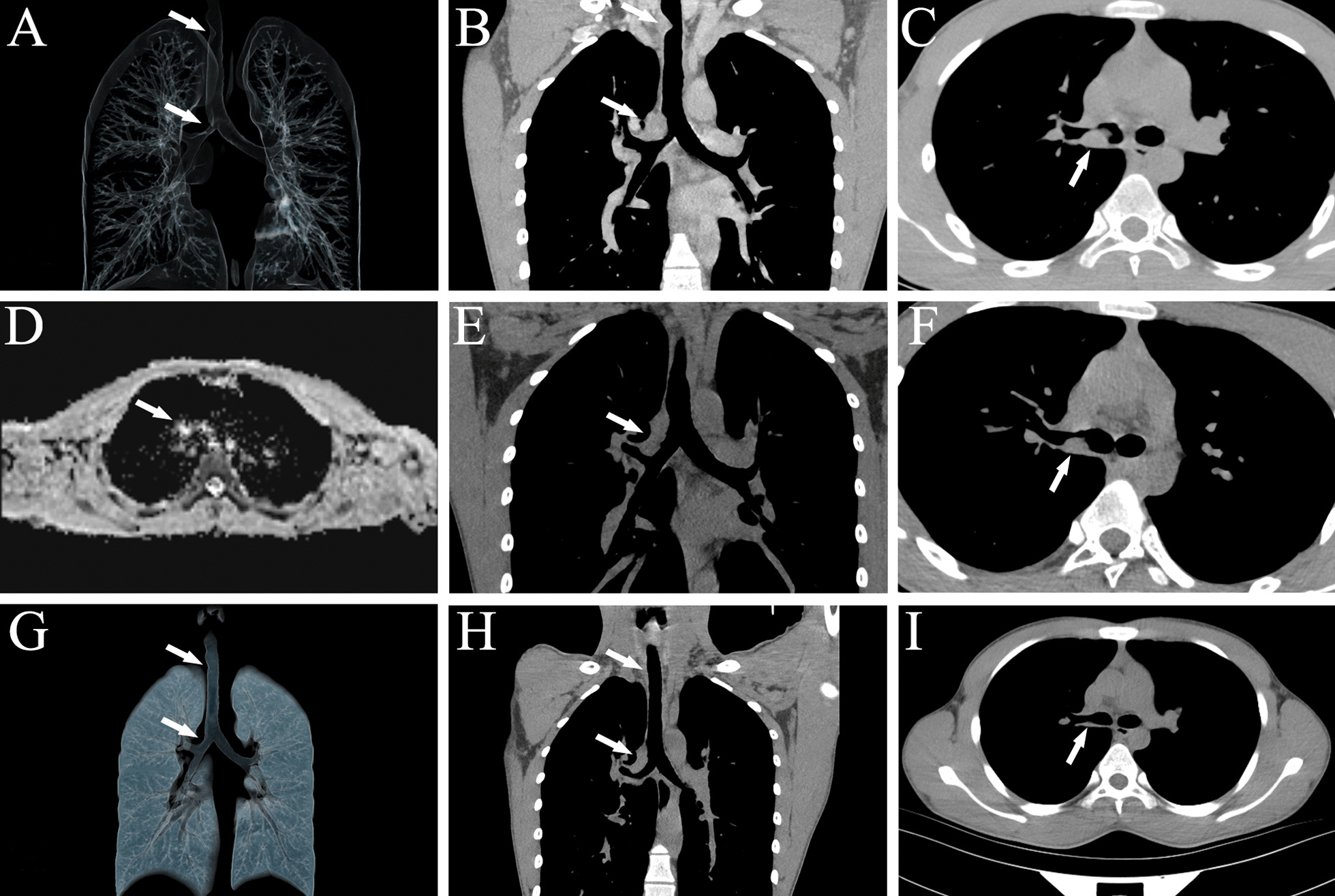
Chest CT image of the RDD patient. **A** Chest CT three-dimensional image showed 0.9 × 1.0 × 1.5 cm irregular neoplasm (arrows) adhered to the right wall of the trachea and 1.0 × 1.4 × 2.5 cm irregular neoplasm (arrows) adhered to the opening of the right main bronchus resulting in airway stenosis. **B** Coronal position. **C** Mediastinal window. **D** DWI of enhanced chest MRI showed that signals from the neoplasms (arrow) in the trachea and the right main bronchus had been enhanced, and the apparent diffusion coefficient value was about 1.7. **E**–**F** The stenosis (arrows) of the trachea and the right main bronchus was significantly improved after two transbronchial resections. **G**–**I** After 5 months of follow-up, the trachea and the right main bronchus were unobstructed (arrows)

**Fig. 2:**
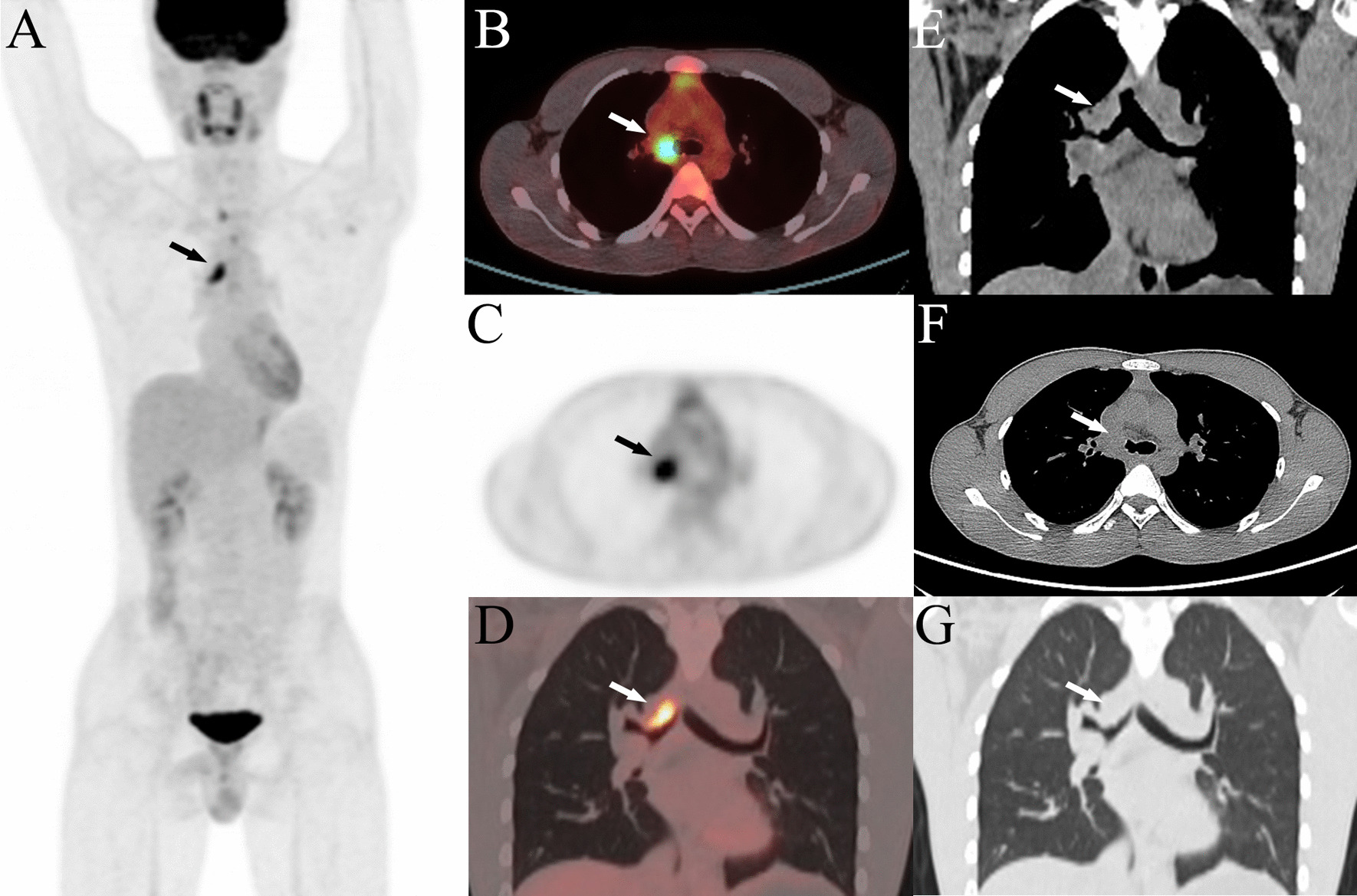
PET/CT image of the RDD patient. Maximum density projection (**A**), transverse mediastinal window (**B**), transverse PET image (**C**) and coronal lung window PET/CT fusion image (**D**) showed high uptake (arrows) of the right main bronchial wall with SUVmax 8.54 and SUVmean 5.08. Coronal mediastinal window (**E**), transverse mediastinal window (**F**) and coronal lung window of CT (G) showed the neoplasm (arrows) in the right main bronchus

On admission in June 2021, vital signs of the patient were body temperature 36.7 °C, pulse rate 84 beats/min, respiratory rate 18 breaths/min, blood pressure 110/70 mmHg and oxygen saturation 98% on room air. On physical examination, whistle sound could be heard in his right lung when inhaling. Tumor biomarkers, such as carcinoembryonic antigen (CEA), neuron-specific enolase (NSE), squamous cell carcinoma associated antigen (SCC), gastrin-releasing peptide precursor (Pro-GRP) and cytokeratin 19 fragment (CYFRA21-1) were normal. Lactate dehydrogenase (LDH), serum ferritin (SF), β2 microglobulin (β2-MG), autoantibody series, anti-neutrophil cytoplasmic antibody (ANCA), antistreptolysin O (ASO), hypersensitive C-reactive protein (hs-CRP), rheumatoid factor (RF), IgA, IgM, total IgG, IgG1, IgG2, IgG3, IgG4, IgE, complement C3, complement C4, κ light chain, γ light chain, lymphocyte subsets (including T cells, B cells and NK cells) were all normal. Blood cell counts, procalcitonin (PCT), interleukin 6 (IL-6) and serum amyloid A (SAA) were also in normal range. Xpert MTB/RIF, acid-fast staining and tuberculosis-DNA-polymerase chain reaction (TB-DNA-PCR) in sputum were also negative. Serum T-SPOT. TB was also negative. Only serum rubella virus IgG (Rub-IgG), cytomegalovirus IgG (CMV-IgG) and herpes simplex virus type I IgG (HSV-I-IgG) were detected positive. Arterial blood gas analysis on room air were in normal range: pH 7.404, PO_2_ 96.5 mmHg, PCO_2_ 35.5 mmHg, HCO_3_^−^ 21.7 mmol/L, SO_2_ 97.4%. Chest CT three-dimensional image of trachea and bronchial showed 0.9 × 1.0 × 1.5 cm irregular neoplasm adhered to the right wall of the trachea at the first thoracic vertebra level and 1.0 × 1.4 × 2.5 cm irregular neoplasm adhered to the opening of the right main bronchus (Fig. 1A–C). Diffusion-weighted imaging (DWI) of enhanced chest magnetic resonance (MRI) showed that signals from the neoplasms in the trachea and the right main bronchus had been enhanced, and the apparent diffusion coefficient (ADC) value was about 1.7, which indicated malignant (Fig. 1D). Positron emission tomography/computed tomography (PET/CT) showed high uptake of the lesions with maximum standardized uptake value (SUV max) 8.54 and mean standardized uptake value (SUV mean) 5.08. There were no obviously enlarged lymph nodes in bilateral hilum and mediastinum (Fig. 2). That means we did not exactly confirm the lesions were malignant or not. Nasopharyngoscope showed no abnormalities.

**Fig. 3:**
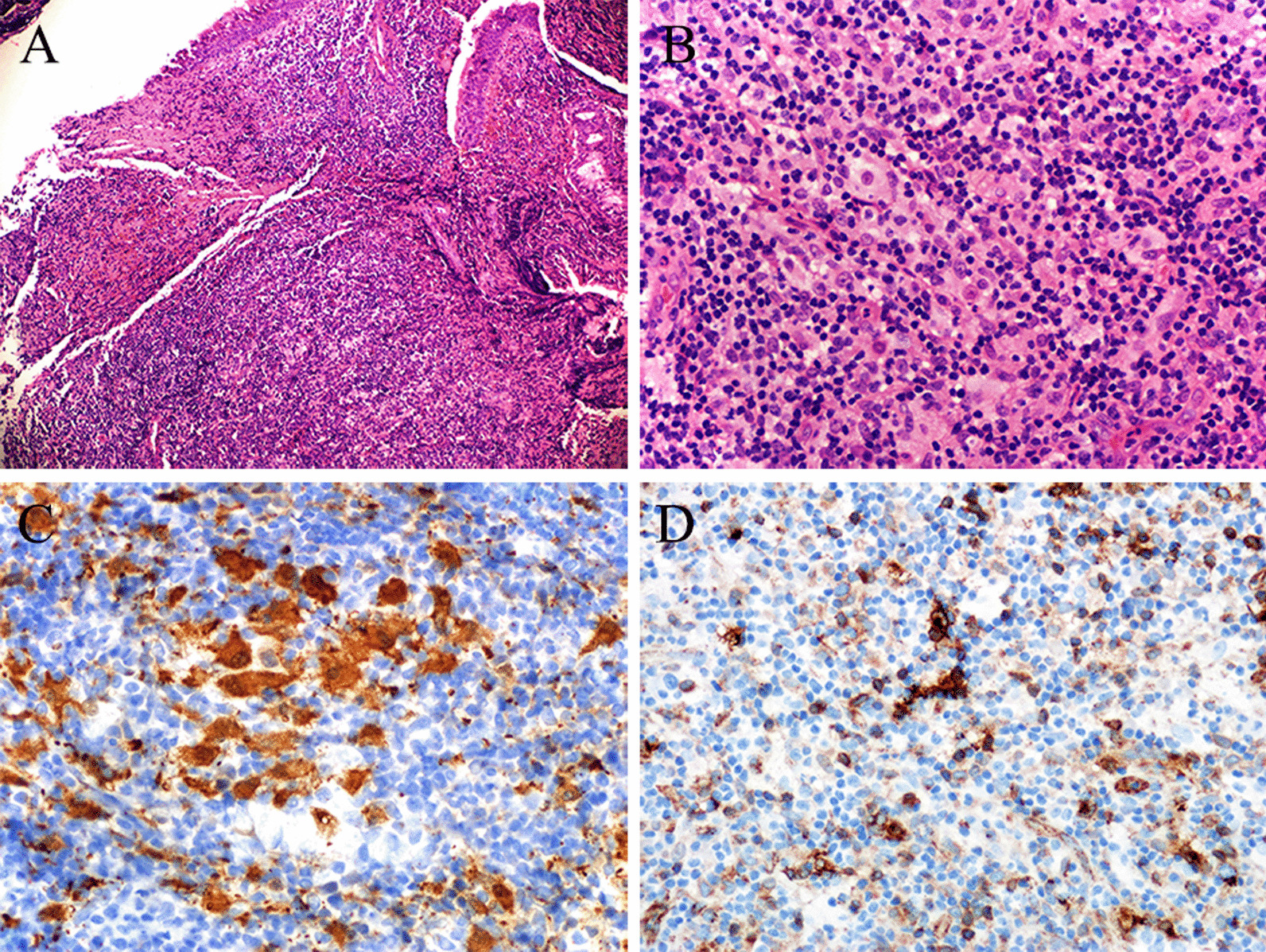
Histopathological and immunohistochemical characteristics of the RDD patient. **A** Numerous lymphocytes, plasma cells, neutrophils and a few eosinophils infiltrated in the bronchial submucosa (HE 10 × 10). **B** A few large histocytes were scattered in the mass (HE 10 × 40). **C** The large histocytes showed S-100 protein positive with emperipolesis phenomenon (IHC × 400). **D** HLA-DR positive in the large histiocytes (IHC × 400)

After admission, transbronchial mass biopsy via flexible fiber scope (Olympus BF-IT260) was performed twice and the masses were removed by directly clamping through large biopsy forceps at the same time. The histopathological, immunohistochemistry (IHC), special staining, molecular pathology and metagenomic next-generation sequencing (mNGS, DNA + RNA) of both biopsy samples were similar. Numerous lymphocytes, plasma cells, neutrophils and a few eosinophils infiltrated the bronchial submucosa (Fig. 3A). A few large histocytes were scattered in the mass (Fig. 3B). Special staining showed PAS (−), acid fast (−) and silver hexamine (−).IHC showed ALK (5A4) (−), CD1a (−), CD2 ( +/−), CD20 (−/ +), CD3 ( +/−), CD30 (−), CD4 ( +/−), CD5 ( +/−), CD56 (−), CD7 ( +/−), CD8 (+/−), CK5/6 (−), Cyclin D1 (+ , scattered), CMV (−), Langerin (−), GATA-3 (−), MUM-1 (+ , scattered), PAX5 (+ , local), TDT (−), TTF-1 (−). S-100 (+ , scattered) (Fig. 3C), CD68 (KP1) (+), CD68 (PG-M1) (+ , foci), and HLA-DR ( +) (Fig. 3D). Ki-67 proliferation index was about 40%. Epstein-Barr Virus-encoded RNA (EBER) in situ hybridization was negative. BRAF V600E mutation was not detected by fluorescence PCR. Molecular pathology showed TB-DNA-PCR negative. No malignant tumor cells were detected in bronchoalveolar lavage fluid (BALF) from the right upper lobe. Fungal smear, acid-fast staining, TB-DNA-PCR and bacterial culture of BALF were all negative. TB-DNA-PCR and Xpert MTB/RIF of the masses were also negative. No pathogen was detected in this two biopsy samples by mNGS.

**Fig. 4:**
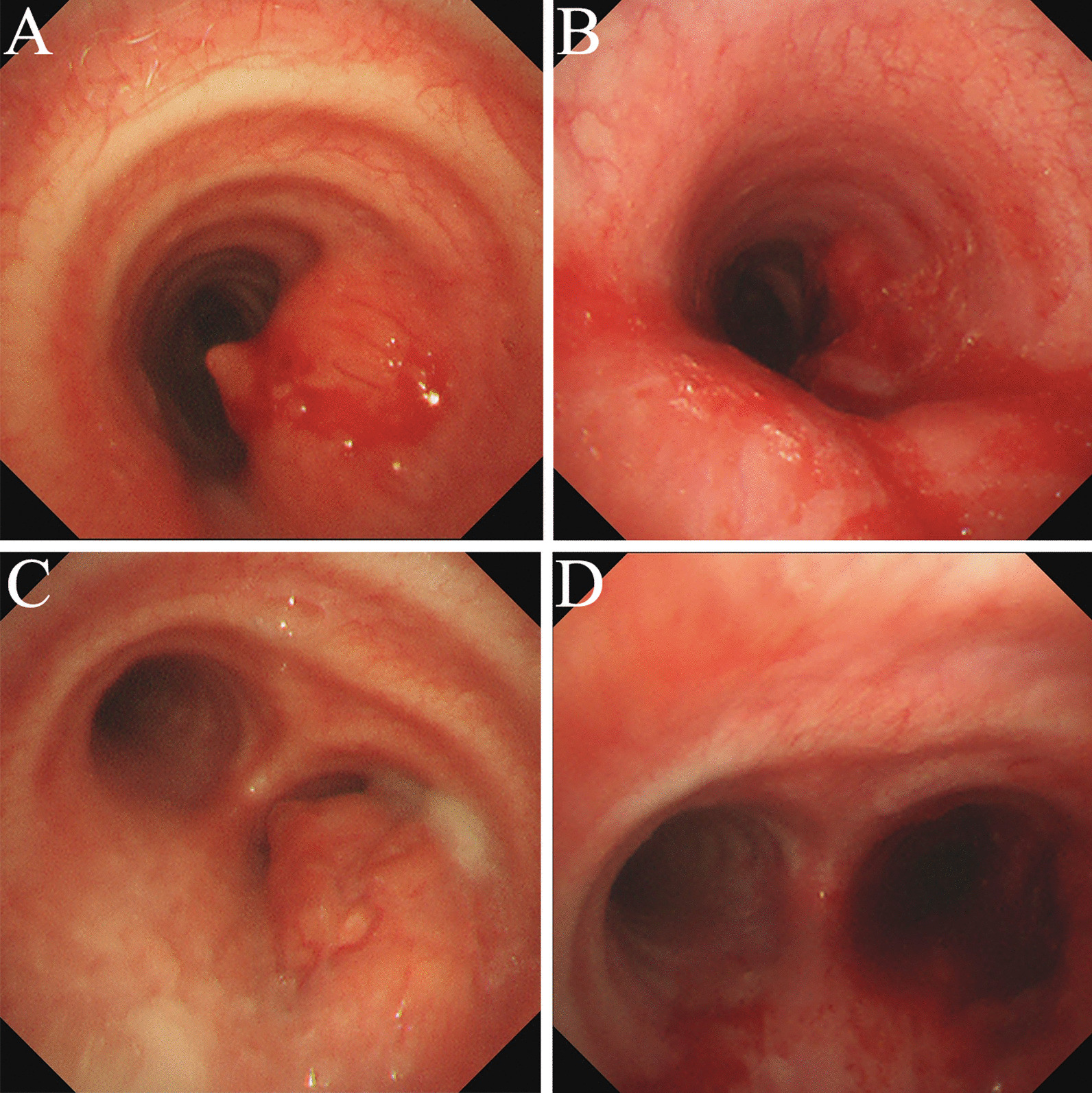
Bronchoscopic findings of the RDD patient. **A** Intratracheal neoplasm resulted in about 40% stenosis at the middle 1/3 segment of the trachea. **B** After two transbronchial resections, the tracheal stenosis was decreased to about 20%. **C** The neoplasm in the opening of the right main bronchus resulted in about 90% of stenosis. **D** After two transbronchial resections, the airway stenosis was decreased to about 10%

According to the examination results above, the patient was diagnosed with primary diffuse RDD in central airway definitely through multidisciplinary discussion of respirology, radiology, oncology, pathology, radiotherapy and thoracic surgery. After two transbronchial resections [2], chest CT and bronchoscopy showed the neoplasms in the trachea and the opening of the right main bronchus were significantly smaller than before, and the airway stenosis was improved significantly (Figs. 1E, F and 4). Cough, shortness of breath of the patient were disappeared, and there was no whistle sound. No more treatment was given to the patient. After 5 months of follow-up, the patient had no symptoms and the trachea and the right main bronchus were unobstructed. (Fig. 1G–I).

## Discussion and conclusions

RDD is a rare histiocytic disease which was firstly described by Destombes in 1965 [3], and later described by Rosai and Dorfman in 1969 as sinus histiocytosis with massive lymphadenopathy which was classified as non-langerhans cell histiocytosis by the Working Group of the Histiocyte Society in 1987 [4, 5]. Classical lymph node type is the most common type of RDD, accounting for 30–50% of RDD patients [1, 6]. Extranodal RDD mainly affects skin (50%), central nervous system (10%), retroorbital (5%), bone (15%), retroperitoneal (5−10%) and upper respiratory tract (10−20%) [1]. Intra-thoracic RDD accounts for only 2% of all RDD patients, which involving the trachea is even rarer [7]. Only 18 cases involving the trachea has been reported until now [7–24]. In addition, only 7 cases were both diffused and involved in central airway with intraluminal mass[7, 8, 10, 12, 14, 20, 24]. By thorough literature search in PUBMED, we believe this is the first report about primary diffuse RDD only involving the central airway and have the risk for suffocation.

Little is known about the etiology and pathogenesis of RDD. It was previously thought to be a reactive, nonneoplastic and nonclonal histological lesion. Although RDD exhibits some characteristics of malignant disease, it is not hyperproliferative [25]. Mutations of ARAF, MAP2K1, NRAS and KRAS were detected both classical and extranodal RDD patients [26, 27]. BRAF V600E mutation has been detected and reported in literature recently, suggesting the disease might be clonal [28–31]. But in this case, BRAF V600E mutation was not detected by fluorescence PCR. Some studies suggested the pathogenesis maybe associated with virus infections, such as human herpes virus-6 (HHV-6), parvovirus B19 or Epstein-Barr virus (EBV), but none of these had been confirmed [25]. Although serum Rub-IgG, CMV-IgG and HSV-I-IgG were positive, tissue mNGS, immunohistochemical EBER and CMV-DNA tests were all negative, which did not confirm the role of the virus in the pathogenesis of RDD.

Since central airway RDD was extremely rare and similar to malignant tumor in imaging and bronchoscopy features, it is difficult to differentiate it from primary airway malignant tumor. The precise diagnosis of central airway RDD depended on pathology and immunohistochemistry. Using Chest CT three-dimensional image can easily figure out the location of the lesion, the depth of invasion, the relationship with peripheral vessels, and provide diagnostic basis for the lesions. In this patient, tracheal and right main bronchial lesions showed high SUV values in PET/CT, which was consistent with the report of Ali et al. [13], Shingo et al. [16], Dong Yujie et al. [18] and Ni Ming et al.[23]. These results indicated that PET/CT had no specificity for the diagnosis of RDD, but systemic PET/CT could clarify whether RDD lesions were limited in a single area or involved in multiple organs. So systemic PET/CT had incomparable value for system evaluation and therapeutic effect evaluation of RDD.

The pathological features of RDD in previous reports showed that different morphologies of hyperproliferative histiocytes with weak staining of nuclear, pale cytoplasm, S-100 (+), CD68 (+), CD163 (+), CD14 (+), HLA-DR (+), CD1a (−) and Langerin (−) by immunohistochemistry [6]. In this case, the pathological features of the lesions were consistent with the above results, and no more lesions were found in the whole body by PET/CT. So the patient was diagnosed with primary diffuse RDD in central airway. RDD combined with IgG4-related diseases had been reported in a few literatures in recent years [20, 32–34], but so far there is no report about primary central airway RDD combined with IgG4-related diseases. In this case, serum IgG4 was in normal range. No evidence of IgG4-related diseases was found by immunohistochemistry.

Sporadic RDD was usually self-limited, with spontaneous remission in up to 50% of cases reported in literature and had a good prognosis. However, up to 10% of patients may die of direct complications, infections or amyloidosis [25]. Asymptomatic cutaneous RDD without enlarged lymph node only need to be followed up. Surgical excision was recommended for patients in unifocal extranodal RDD or for symptomatic airway, cranial, spinal or sinus disease. Systemic therapies, such as glucocorticoid, sirolimus, radiotherapy, chemotherapy and immunomodulators, were recommended for RDD of multifocal and unresectable extranodal lesions. However, there was no standardized regimen currently in this field yet [25]. Some tumor-related driver gene mutations could be detected in a few patients with RDD. The consensus was that NGS for Mitogen-Activated Protein Kinase (MAPK) mutations should be recommended, and targeted therapy should be considered according to driver gene mutations were identified or not. It was reported that 18 patients with central airway RDD were followed up for 3 to144 months. These patients received various treatments, including surgical resection, transbronchial resection, laser resection, glucocorticoid, chemotherapy. Among the 6 patients with surgical resection [14, 15, 18, 19, 22, 24], only 1 patient with diffuse airway RDD relapsed after single lesion resection combined with hormone therapy [14], the rest 5 patients achieved complete response. The other 2 patients with limited airway RDD relapsed after treatments [17, 21], and 3 patients with diffuse airway RDD relapsed all [8, 10, 14]. One patient with diffuse central airway RDD with lymph node and cutaneous lesions died soon after diagnosis [12]. The rest 6 patients received no therapy [7, 11–13, 16, 20]. In this case, the patient had multifocal and unresectable diffuse lesions in central airway with a risk for suffocation. After two transbronchial resections, the patient had no cough, shortness of breath and whistle sound. The stenosis of trachea and the right main bronchus were significantly improved and kept unobstructed for 5 months. These results suggested that transbronchial resection can be the preferred treatment for patients with central airway RDD, due to the less adverse events and better prognosis. We believe this is helpful for clinicians to treat central airway RDD in future.

Briefly, the patient presented with paroxysmal cough, shortness of breath and whistle sound in this report. The features examined by enhanced chest CT, PET/CT, DWI of enhanced chest MRI and bronchoscopy indicated to be malignant tumor. However, RDD was confirmed by multiple transbronchial biopsies and immunohistochemistry definitely. After two transbronchial resections, the airway stenosis improved significantly, the patient's symptoms and signs were significantly reduced. Based on clinical experience and review of the literature, we believed that transbronchial resection was effective and safe for patients with diffuse RDD in central airway.

## Data Availability

The datasets used and analyzed during the current study are available from the corresponding author on reasonable request.

## Abbreviations

RDD: Rosai-Dorfman disease
CT: Computed tomography
PET/CT: Positron emission tomography/computed tomography
DWI: Diffusion-weighted imaging
MRI: Magnetic resonance imaging
FEV_1_: Forced expiratory volume in 1 s
FVC: Forced vital capacity
DLCO: Diffusing capacity for carbon monoxide
MVV: Maximal voluntary ventilation
CEA: Carcinoembryonic antigen
NSE: Neuron-specific enolase
SCC: Squamous cell carcinoma associated antigen
Pro-GRP: Gastrin-releasing peptide precursor
CYFRA21-1: Cytokeratin 19 fragment
LDH: Lactate dehydrogenase
SF: Serum ferritin
β2-MG: β2 Microglobulin
ANCA: Anti-neutrophil cytoplasmic antibody
ASO: Antistreptolysin O
hs-CRP: Hypersensitive C-reactive protein
RF: Rheumatoid factor
PCT: Procalcitonin
IL-6: Interleukin 6
SAA: Serum amyloid A
TB-DNA-PCR: Tuberculosis-DNA-polymerase chain reaction
Rub-IgG: Rubella virus IgG
CMV-IgG: Cytomegalovirus IgG
HSV-I-IgG: Herpes simplex virus type I IgG
ADC: Apparent diffusion coefficient
SUV: Standardized uptake value
EBER: Epstein-Barr Virus-encoded RNA
BALF: Bronchoalveolar lavage fluid
mNGS: Metagenomic next-generation sequencing

## References

[1] Goyal G, Young JR, Koster MJ, Tobin WO, Vassallo R, Ryu JH, Davidge-Pitts CJ, Hurtado MD, Ravindran A, Sartori Valinotti JC (2019). The mayo clinic histiocytosis working group consensus statement for the diagnosis and evaluation of adult patients with histiocytic neoplasms: erdheim-chester disease, langerhans cell histiocytosis, and Rosai-Dorfman disease. Mayo Clinic Pro.

[2] Jin F, Li Q, Li S, Wang H, Bai C, Zeng Y, Zhou R, Cai Z, Chen L, Feng J (2019). Interventional bronchoscopy for the treatment of malignant central airway stenosis: an expert recommendation for China. Respiration.

[3] Destombes P (1965). Adenitis with lipid excess, in children or young adults, seen in the Antilles and in Mali. (4 cases). Bull Soc pathol Exot Filiales.

[4] Rosai J, Dorfman RF (1969). Sinus histiocytosis with massive lymphadenopathy. A newly recognized benign clinicopathological entity. Arch Pathol.

[5] Histiocytosis syndromes in children (1987). Writing group of the histiocyte society. Lancet.

[6] Emile JF, Abla O, Fraitag S, Horne A, Haroche J, Donadieu J, Requena-Caballero L, Jordan MB, Abdel-Wahab O, Allen CE (2016). Revised classification of histiocytoses and neoplasms of the macrophage-dendritic cell lineages. Blood.

[7] Boissière L, Patey M, Toubas O, Vella-Boucaud J, Perotin-Collard JM, Deslée G, Lebargy F, Dury S (2016). Tracheobronchial involvement of rosai-dorfman disease: case report and review of the literature. Medicine.

[8] Carpenter RJ, Banks PM, McDonald TJ, Sanderson DR (1978). 3rd Sinus histiocytosis with massive lymphadenopathy (Rosai-Dorfman disease): report of a case with respiratory tract involvement. Laryngoscope.

[9] Courteney-Harris RG, Goddard MJ (1992). Sinus histiocytosis with massive lymphadenopathy (Rosai-Dorfman disease): a rare case of subglottic narrowing. J Laryngol Otol.

[10] Ahsan SF, Madgy DN, Poulik J (2001). Otolaryngologic manifestations of Rosai-Dorfman Disease. Int J Pediatr Otorhinolaryngol.

[11] Ottaviano G, Doro D, Marioni G, Mirabelli P, Marchese-Ragona R, Tognon S, Marino F, Staffieri A (2006). Extranodal Rosai-Dorfman disease: involvement of eye, nose and trachea. Acta Otolaryngol.

[12] Cherif J, Toujani S, Mehiri N, Louzir B, Kchir N, Beji M (2008). Rosai and Dorfman disease with pleural involvement: case report. Sci World J.

[13] Ali A, Mackay D (2009). Rosai-Dorfman disease of the lung. Thorax.

[14] Xie BS, Li RH, Yue WX, Zheng GY, Jin L (2009). Sinus histiocytosis with massive lymphadenopathy(Rosai-Dorfman disease)of the airway:a case report and review of the literature. Zhonghua Jie He He Hu Xi Za Zhi.

[15] Zhou LF, Chen L, Zhu Q, Wang C, Xu H, Cui XF, Jiang LF, He SH, Huang M, Yin KS (2010). Unusual life-threatening Rosai-Dorfman disease of the trachea: role of NF-κB. Thorax.

[16] Noguchi S, Yatera K, Shimajiri S, Inoue N, Nagata S, Nishida C, Kawanami T, Ishimoto H, Sasaguri Y, Mukae H (2012). Intrathoracic Rosai-Dorfman disease with spontaneous remission: a clinical report and a review of the literature. Tohoku J Exp Med.

[17] Syed A, Malhotra R, Shojaee S, Shepherd RW (2013). Exophytic tracheal mass. A rare presentation of Rosai-Dorfman disease. Ann Am Thorac Soc..

[18] Dong YJ, Mu J, Cai YR, Zhou SJ, Zhang HQ (2013). Primary sinus histiocytosis of the trachea:a case report and review of literature. Zhonghua Jie He He Hu Xi Za Zhi.

[19] Sun WM, Li W, Liu ZF, Zheng Y (2015). One case: tracheal Rosai-Dorfman disease. J Pract Radiol.

[20] Apperley ST, Hyjek EM, Musani R, Thenganatt J (2016). Intrathoracic Rosai Dorfman disease with focal aggregates of IgG4-bearing plasma cells. Case report and literature review. Ann Am Thorac Soc..

[21] Uzunhan Y, Chabrol A, Kambouchner M, Martinod E (2018). Bronchial Involvement in Rosai Dorfman Disease. Ann Thorac Surg.

[22] Santosham R, Santosham R, Jacob SS, Phadke AU, Ponduru T (2019). Rosai-Dorfman disease of the trachea: an extremely rare benign tumor. Asian Cardiovasc Thorac Ann.

[23] Ni M, Xie Q, Zhou HC (2019). ^18^F-FDG PET/CT imaging in tracheal Rosai-Dorfman Disease: a case report. Chin J Nucl Med Mol Imaging..

[24] Al-Maghrabi H, Elmahrouk A, Feteih M, Jamjoom A, Al-Maghrabi J (2020). Rosai-Dorfman disease with pulmonary involvement mimicking bronchogenic carcinoma. J Cardiothorac Surg.

[25] Bruce-Brand C, Schneider JW, Schubert P (2020). Rosai-Dorfman disease: an overview. J Clin Pathol.

[26] Diamond EL, Durham BH, Haroche J, Yao Z, Ma J, Parikh SA, Wang Z, Choi J, Kim E, Cohen-Aubart F (2016). Diverse and Targetable kinase alterations drive histiocytic neoplasms. Cancer Discov.

[27] Garces S, Medeiros LJ, Patel KP, Li S, Pina-Oviedo S, Li J, Garces JC, Khoury JD, Yin CC (2017). Mutually exclusive recurrent KRAS and MAP2K1 mutations in Rosai-Dorfman disease. Mod Pathol.

[28] Dufour J, Mathon B, Touat M, Hoang-Xuan K, Mokhtari K, Idbaih A (2021). BRAF mutation in overlapping form of Erdheim-Chester and Rosai Dorfman diseases: a unique case restricted to the central nervous system. Rev Neurol.

[29] Richardson TE, Wachsmann M, Oliver D, Abedin Z, Ye D, Burns DK, Raisanen JM, Greenberg BM, Hatanpaa KJ (2018). BRAF mutation leading to central nervous system rosai-dorfman disease. Ann Neurol.

[30] Fatobene G, Haroche J, Hélias-Rodzwicz Z, Charlotte F, Taly V, Ferreira AM, Abdo ANR, Rocha V, Emile JF (2018). BRAF V600E mutation detected in a case of Rosai-Dorfman disease. Haematologica.

[31] Mastropolo R, Close A, Allen SW, McClain KL, Maurer S, Picarsic J (2019). BRAF-V600E-mutated Rosai-Dorfman-Destombes disease and Langerhans cell histiocytosis with response to BRAF inhibitor. Blood Adv.

[32] Chen LYC, Slack GW, Carruthers MN (2021). IgG4-related disease and Rosai-Dorfman-Destombes disease. Lancet.

[33] Iyengar NS, Golub D, McQuinn MW, Hill T, Tang K, Gardner SL, Harter DH, Sen C, Staffenberg DA, Thomas K (2020). Orbital Rosai-Dorfman disease initially diagnosed as IgG4-related disease: a case report. Acta Neuropathol Commun.

[34] Tracht J, Reid MD, Xue Y, Madrigal E, Sarmiento JM, Kooby D, Alese OB, Krasinskas AM (2019). Rosai-Dorfman disease of the pancreas shows significant histologic overlap With IgG4-related disease. Am J Surg Pathol.

